# Supplementary material for: Ten minutes of transcranial static magnetic field stimulation does not reliably modulate motor cortex excitability
Source: PLoS One. 2020 May 26;15(5):e0233614. doi: 10.1371/journal.pone.0233614 (PMC7250443; doi:10.1371/journal.pone.0233614)
Supplement: Attachment — Submitted filename: Response_to_Reviewers_final.docx [file pone.0233614.s002.docx]

Reviewer #1: PONE-D-19-34372

Lorenz et al. investigated influence of tSMS on excitability of the primary motor cortex (M1). This is in essence a negative study pointing null effect of tSMS, regardless of inclusion of the oddball task; the only significant change was observed when the order of experimental sessions was taken into consideration. I believe null effects should be made public, as long as the methodology was rigorous and sound. In this regard, I have the following concerns.

1) Initially, the sample size had been calculated based on a certain medium effect size of f = 0.25 (page 5). What if this assumption was not the case? Did the authors calculate? If the actual effect size was smaller, more participants would be needed to detect a difference.

Indeed, if the effect size would have been smaller, the number of participants needed would be higher. Unfortunately, in most of the previous reports on tSMS effects on motor cortex excitability (Oliviero et al., 2011; Silbert et al., 2013, Dileone et al., 2018) no effect size was reported. Thus, our power estimation was based on an assumed medium effect size of 0.25 (following Cohen in case of F-statistics). With an alpha error of 0.05 and a power of 0.8, for a 2 x 7 within factor design the calculated sample size is 18. We recruited 24 subjects and were lucky to be able to analyze exact 18 subjects.

However, in most of the previous studies less subjects have been investigated (Oliviero et al., 2011: 11 subjects per experiment; Silbert et al., 2013: 10 subjects, Nojima et al., 2015: 10/10/10 per group; Dileone et al., 2018: 10/10/9/14(but 8 real 8 sham)/18(but 9 real 9 sham) for the 5 experiments, respectively). All cited studies demonstrated significant modulatory effects of tSMS. Therefore, it seems unlikely that our sample size was just too small to detect possible changes.

Furthermore, Nojima et al., 2015 reported an effect size of η^2^= 0.163 for the Time*Group Interaction and of η^2^= 0.306 in the post hoc analysis for the significant suppression of MEP in the post-0 measurement, directly after tSMS exposure. Both values represent a high effect size, following Cohen (1988) who classified effect sizes above 0.14 as high. From that our assumption of a medium effect size is a conservative estimation.

2) The significant effect of SESSION (first, second)*TIME interaction was observed in the second ANOVA with assumed sphericity. I understand this ANOVA was a (so to speak) post-hoc one; nevertheless, a three-way ANOVA could have been conducted first, using SESSION (with and without oddball), ORDER (first, second), and TIME as factors.

Indeed, a three-way ANOVA including the factors SESSION (first, second, your “ORDER”), INTERVENTION (without and with oddball, your “SESSION”), and TIME can be conducted. See the analysis here:

Please note that in the manuscript, we used the factor named SESSION both for intervention type as well as for order.

In this 3-way ANOVA the group of 18 subjects is splitted so that only 9 subjects are averaged for the factors SESSION and INTERVENTION, reducing the power compared to the approach presented in the manuscript. Furthermore, since our aim was to investigate the influence of the oddball-task on MEP Amplitude (i.e. SESSION (intervention)*TIME), we think that a 3-way ANOVA is not appropriate as first statistical attempt. The question of the influence of session order was a post-hoc question which should not be addressed in the first place. Therefore, we feel that the way we present the data is the appropriate one in a scientific sense.

3) As a part of conclusion reads “absence of evidence is not evidence of absence (page 17).” I completely agree this statement, but just after that “Further studies with adequate statistical power” were claimed. We might not be able to say something about absence of evidence based on studies without adequate statistical power.

Thank you for this sophisticated hint. Indeed, without adequate statistical power neither evidence nor the absence of evidence can be stated. This does not touch the core problem, i.e. the generation of evidence of absence, which cannot be reached regardless the adequacy of power.

We changed the passage in the conclusion section. It now reads: ”However, these results do not exclude a tSMS effect in general. Further studies with adequate statistical power and within-subject replication would clarify this general question.”

4) In Abstract, the authors argued habituation can be a key factor explaining the results, but this idea was not explicitly dealt in Discussion.

Thank you for indicating this discrepancy. It was not in our intention to postulate habituation as a key factor explaining our results. In the abstract, we only stated: “there might be a habituation effect to the whole procedure, resulting in less variability when subjects underwent the same experiment twice.”

We tried to include the habituation argument in this passage of the discussion: “Recently, for intermittent theta burst stimulation (iTBS) it was suggested that the modulatory effect might habituate if the same procedure was repeated [24]. In their study, the increase of corticospinal excitability following iTBS was present in a first session, but disappeared when a second, identical session was followed about one week apart. The authors concluded that there is a high interindividual variability as well as a low intraindividual reliability of iTBS effects, and that group results based on only one session should be interpreted with caution [24].” (page 15f.).

Since the difference between session 1 and 2 in our study is mainly driven by a subsample of 4 subjects (see Fig.3), a putative habituation on the whole procedure plays more a secondary role and was therefore not dealt with more attention. In fact, individual variability in MEP amplitudes seems to be smaller in the subject´s second sessions (which can be seen in the smaller standard errors in Fig. 2 as well), and we just argued that this might be due to a kind of habituation to the procedure.

5) (Minor) sagittal “plain (page 6)” should be sagittal “plane.”

We corrected this spelling mistake.

Reviewer #2: Review MS Nr. Pone-D-19-34372
This sham-controlled TMS-study was planned in order to examine the influence of cognitive activity (auditory oddball task) on tSMS- induced cortico-spinal excitability (CSE) changes. CSE was assessed via single pulse motor evoked potentials (MEP) in a pre- post experimental design. The results show that a period of 10 min tSMS (sham) did not modulate CSE, and there was no co-modulation from cognitive activity. The authors suggested that fluctuations in MEP amplitude could have masked possible weak modulatory effects. The study has a rationale and is clearly stated. However there are some points limiting its scientific value.

As described in the introduction, this study is a repetition an earlier TMS-study using 10 min tSMS (Kufner et al. 2017). Since after tSMS no effect was found in the earlier study, it is unlikely to find robust tSMS stimulation effects that are useful to clarify the additional influence of cognitive activity. In the introduction of this study I found no discussion how cognitive activity may interact with simultaneous tSMS with respect to pre-post-changes in motor cortical excitability and the formation neuroplasticity.

Thank you for this request. We tried to explain the putative interaction between tSMS and cognitive activity introduced by the auditory oddball task. In the introduction, we wrote:

"It was suggested that the use of an oddball task could offer an explanation for the difference in results [17]. Counting mentally could activate the motor cortex due to individual finger counting habits [18], and these habits might manipulate the structure of mental number representations [19]. It has also been observed that productive and perceptive linguistic tasks increase motor cortex excitability bilaterally, indicating that forming number words mentally while counting could cause activation in the hand representation of the motor cortex [20]." (page 3f.)

We now added the following sentence: "Activation of the motor cortex without motor output might therefore interfere with neuroplastic modulation induced by static magnetic fields."

We think that this passage now clarifies both, the putative interaction of tSMS in the motor system and a cognitive activity involving motor cortex as well as the motivation for the study.

The reviewer is right with the notion that we did not find a tSMS effect in our previous study (Kufner et al. 2017). However, the problem here was that we only measured in combination with the auditory oddball task. Therefore, indeed, this cognitive task could have interacted with the modulatory influence of tSMS, destroying the putative tSMS effect. This exactly was postulated in a letter by Foffani et al. (2017). We cannot follow the argument of the reviewer that is unlikely to find robust tSMS effects in the light of our previous results (Kufner et al. 2017), since a replication of the study by Oliviero et al. (2011), without any additional cognitive task, should yield tSMS effects.

Although tSMS is described as a new simple form of inhibitory NIBS, and was highlighted a promising tool for brain stimulation, the mechanisms behind the neuromodulatory effects still remain unclear. Reorientation of diamagnetic anisotropic plasma membrane phospholipids (Rosen AD, 2003), coupling of mechanically-activated ion channels to ferromagnetic particles (Dobson et al. 1996) and cryptochromes (Landler & Keays 2018) are in discussion. Thus it should be stated somewhere that the fundamental research on tSMS is still at the beginning, and from this point it appears speculative to speak about treatment of neurological or psychiatric diseases.

We agree that fundamental research on tSMS is at the beginning. Since other groups already published applications of tSMS in the context of neurological diseases, we stated in the introduction: " An effect of tSMS on cortical excitability comparable to those evoked by other noninvasive brain stimulation methods such as repetitive TMS [8-10] or transcranial direct current stimulation [11-13], but without the side effects [14, 15] would offer a wide range of possibilities for, i.e., treatment of neurological or psychiatric diseases." (page 3). However, in order to clarify our view on the early state of tSMS research, we added the following passage in the discussion: “Although there are first attempts for a potential treatment of neurological or psychiatric diseases using tSMS [41], the fundamental research of tSMS is still just at the beginning.”(page 17).

Experimental part. The TMS-assessments for such a study are rather minimalistic. To gain more insight into the formation motor cortical plasticity and metaplasticity (tSMS + task) assessments of intracortical circuits by using by paired pulse TMS (SICI, SICF) showed advantageous (Dileone et. al. 2018). In the current study CSE was probed at a stimulation intensity to produce 1 mV MEPs. Here graded stimulation intensities resulting in MEP recruitment curves would provide information about the excitability changes of cortico-spinal circuits over a wider input-output range.

We agree that besides MEP amplitude other parameters are suitable for measuring changes in cortico-spinal excitability. Since paired pulse TMS paradigms (SICI, ICF) show only moderate to poor test-retest-reliability whereas that for MEP amplitudes is better (i.e. Hermsen et al., 2016, J Neurol Sci 362), those measurements may not be that suitable to detect such small changes possibly caused by tSMS, although there was an effect reported by Dileone et al., 2018. However, all published data so far report a suppression effect of tSMS on MEP amplitude. Furthermore, the aim of the present study was explicitly to clarify the issue of putative interference of a cognitive task which was hypothesized to cancel the tSMS effect. Therefore, we chose MEP amplitude as the only dependent variable.

At least an exposure period (10 minutes tSMS) not appears optimal for such a study. Studies have shown that 10 minutes is the minimum, more stable and more long-lasting effect were obtained after periods of 20 and 30 min tSMS. From this point of view it is recommended to repeat the experiments with a longer stimulation period.

We agree with the reviewer that a longer exposure of tSMS might increase the modulatory effect, as recent publications demonstrate. However, the aim of the study was to replicate the original finding, as well as to answer the question concerning the impact of the additional cognitive task, as raised by Foffani and Dileone (2017). Therefore, we did not prolong the exposure time. The main question of the study, namely whether the inhibitory effect of 10min tSMS reported by Oliviero et al., 2011 is abrogated if an acoustic oddball task was used during stimulation, would not be answered by using a prolonged stimulation time.

Minor points:
The title is no very representative for a study focusing on the influence of cognitive activity

Thank you for this claim. Indeed, the title would not be appropriate for a study focusing on the influence of cognitive activity. However, in the present study we do not focus on the influence of cognitive activity. Instead, we focus on the reliability of putative tSMS effects on motor cortex excitability, trying to replicate former results and to investigate the reasons for replication failures. Therefore, we think that the title focusses on the main aspects of the manuscript.

Fig. 1 and Fig.2: the variations (standard error?) in the pre-assessment blocks are much smaller than in the post-assessment blocks. This is quite uncommon for TMS values, and needs explanation

We show normalized values. The normalization took place on the pre-assessment blocks. If we would average the two pre time points together to one single time point, no variation at all would be found since each and any subject, by definition, would have the value of 1. Only the fact that we kept the time resolution with two aggregation states in the pre-assessment blocks allowed us to include these two time points into the inference analysis.
