# Supplementary material for: Ten minutes of transcranial static magnetic field stimulation does not reliably modulate motor cortex excitability
Source: PLoS One. 2020 May 26;15(5):e0233614. doi: 10.1371/journal.pone.0233614 (PMC7250443; doi:10.1371/journal.pone.0233614)
Supplement: Attachment — Submitted filename: rev2_Phone-D-19-34372R2.docx [file pone.0233614.s003.docx]

The authors only made minor changes in the new version of the manuscript. Not all suggestions were replied, for example the use recruitment curves was recommended in the first review to test pre- post changes in motor cortex excitability (MCE)

The authors stated in their response that “aim of this study was to replicate the original finding…… as rised by Foffani and Dileone (2017)”. Thus the intention of this study was not to evaluate the effect of tSMS on MCE in relation to the previous research in this field. This would include longer stimulation times and additional TMS protocols. However the current title of their study suggests that tSMS per se produces no modulatory effect on MCE.

As solution to this problem a more result oriented title is proposed, for example: “Ten minutes of tSMS does not reliable modulate MCE”

Minor Revision!
