# Supplementary material for: Ten minutes of transcranial static magnetic field stimulation does not reliably modulate motor cortex excitability
Source: PLoS One. 2020 May 26;15(5):e0233614. doi: 10.1371/journal.pone.0233614 (PMC7250443; doi:10.1371/journal.pone.0233614)
Supplement: Attachment — Submitted filename: Response_to_reviewers_R2.docx [file pone.0233614.s004.docx]

Reviewer #1: I would like to thank the authors for the elaboration and revision. The responses to my previous comments are in principle satisfactory. Please include the ideas described in the comments in the main text.

More specifically,

1) Please report the effect size, as well as the p-values. Methods states that the power analysis was performed based on the f-value as an indicator for the effect size, whereas in the response to the comments eta-squared was presented. In the main text, please also be congruent.

Thank you for this hint. For the significant SESSION*TIME interaction in the analysis with respect to session order, we now added the missing effect size as η^2^ value (page 11). Furthermore, we rigorously checked all results of inference statistics reported with respect to completeness.

Since our experiment was based on the first study by Oliviero et al., 2011 without reporting any effect sizes, in our former version of the manuscript we decided to estimate a medium effect size for power analysis, based on the f-value reported. However, due to the fact that in a more recent paper (Nojima et al., 2015) real effect sizes were reported and in line with our own calculation, we now decided to include a power analysis on the basis of the reported η^2^ values instead. We changed the passage in the methods section. It now reads:

“Unfortunately, in the work of Oliviero et al., 2011 no effect size has been reported. Therefore, we based our power estimation (G*Power 3.1.7) on the effect size reported more recently (Nojima et al., 2015). In their analysis on MEP ratio for the left hand, they observed a significant Time by Group- interaction with an effect size of η^2^=0.163. Together with their sample size of n=20 (between- group) and an assumed α-error of 0.05, the power of their results was 0.24. Thus, using those values the calculated sample size for our experiment was 22. We decided to recruit 24 subjects to achieve appropriate power.”

2) Please describe explicitly why the authors considered the three-way ANOVA inappropriate in this study. Without a priori assumption it would be the first analysis.

We included the following passage in the results section:

“Furthermore, a friendly anonymous reviewer encouraged us to report a 3-way ANOVA including the factors SESSION (first/second), INTERVENTION (with/without oddball), and TIME (1-7).

There was a significant effect for the factor SESSION (F_(1,224)_=12.35, p<0.001, η^2^= 0.05). However, there was no other significant effect for any factor (INTERVENTION: F_(1,224)_<0.001, p=0.99; TIME: F_(6,224)_=0.66, p=0.68) nor any statistically significant interaction.

Please note that in this 3-way ANOVA the group of 18 subjects is splitted so that only 9 subjects are averaged for the factors SESSION and INTERVENTION. Furthermore, since our aim was to investigate the influence of the oddball-task on MEP Amplitude (i.e. INTERVENTION*TIME), the question of the influence of session order is a post-hoc question.”

Reviewer #2: The authors only made minor changes in the new version of the manuscript. Not all suggestions were replied, for example the use recruitment curves was recommended in the first review to test pre- post changes in motor cortex excitability (MCE)

Thank you for indicating this. We are sorry for skipping a response to the topic recruitment curves. Indeed, recruitment curves seem to be more informative regarding modulation excitability in the motor system. However, it is more time-consuming to apply recruitment curves with respect to MEP recordings with a fixed intensity. Since we aimed to replicate several previous results, we restricted our dependent variable to MEP amplitudes.

The authors stated in their response that “aim of this study was to replicate the original finding…… as rised by Foffani and Dileone (2017)”. Thus the intention of this study was not to evaluate the effect of tSMS on MCE in relation to the previous research in this field. This would include longer stimulation times and additional TMS protocols. However the current title of their study suggests that tSMS per se produces no modulatory effect on MCE.

As solution to this problem a more result oriented title is proposed, for example: “Ten minutes of tSMS does not reliable modulate MCE”

Since the reviewer is right with his or her argument, we changed the title of the manuscript. It now reads “Ten minutes of transcranial static magnetic field stimulation does not reliably modulate motor cortex excitability”.
